# Changes in peroxisome proliferator-activated receptor alpha target gene expression in peripheral blood mononuclear cells associated with non-alcoholic fatty liver disease

**DOI:** 10.1186/s12944-018-0901-7

**Published:** 2018-11-14

**Authors:** Tian Tian Li, Tian Bi Tan, Hai Qing Hou, Xiao Yun Zhao

**Affiliations:** 1 Medical Experimental Center of Qilu Hospital (Qingdao), Shandong University, Qingdao, 266035 China; 2 Dynacare, 150 Montreal Road, Ottawa, ON Canada; 3 Clinical Lab of Qilu Hospital (Qingdao), Shandong University, Qingdao, 266035 China; 4 Qingdao Key Lab of Mitochondrial medicine, Hefei Road No 758, Qingdao, 266035 China

**Keywords:** NAFLD, Obesity, Insulin resistance, PBMC, PPARα

## Abstract

**Objective:**

To identify differences in the expression of peroxisome proliferator-activated receptor alpha (PPARα) target genes in human peripheral blood mononuclear cells (PBMCs) associated with non-alcoholic fatty liver disease (NAFLD) among Chinese individuals.

**Methods:**

Thirty healthy subjects were selected as the control group (CN), and 43 patients newly diagnosed with NAFLD were subdivided into two groups, non-obese group (NF, *n* = 21) and obese group (OF, *n* = 22). Expression of PPARα and its target genes was determined in PBMCs. The levels of liver cell damage markers, total cholesterol (TC), triglyceride (TG), free fatty acids (FFA), glucose, and insulin were determined in serum.

**Results:**

Compared to the CN group, the blood pressure and homeostasis model assessment for insulin resistance (HOMA-IR) were increased in the other groups (*P* < 0.05), while the systolic blood pressure (SBP) and liver cell damage markers were significantly increased in the OF group (*P* < 0.05). In the OF group, PPARα target gene expression was 2.03–3.31 times higher than that in the CN group, and a negative correlation was found between PPARα target gene expression and abdominal circumference (AC), body mass index (BMI), diastolic blood pressure (DBP). Additionally, solute carrier family 25 (carnitine/acylcarnitine translocase) member 20 (SLC25A20) and acyl-coenzyme A dehydrogenase 2 long chain (ACADVL) were negatively correlated with HOMA-IR; PPARα, acetyl-coenzyme A dehydrogenase 2 (ACAA2), and carnitine palmitoyltransferase 1A (CPT1A) were positively correlated with HOMA-IR.

**Conclusion:**

There is an up-expression of PPARα target genes in the PBMCs of NAFLD patients, possibly leading to changes in β-oxidation and insulin resistance.

## Background

Non-alcoholic fatty liver disease (NAFLD) is a chronic liver lipid metabolism syndrome caused by factors other than alcohol, hepatotoxic drug consumption or liver viruses. It exhibits pathological features such as liver cell steatosis and deposition [1]. The high global incidence of NAFLD is due to its association with obesity and diabetes. The incidence of NAFLD is 20–30% among the general population, 40–70% among obese individuals, 30–50% among diabetic patients, and 90% among hyperlipidemic patients [2–6]. Based on the degree of lipid steatosis, inflammation, and fibrosis within diseased liver tissue, NAFLD is categorized into five stages: hepatic steatosis, nonalcoholic steatohepatitis (NASH), fibrosis, cirrhosis, and cancer [7], with exact pathogenesis being unknown.

Peroxisome proliferator-activated receptors (PPARs) belong to the superfamily of nuclear hormone receptors that include PPARα, PPARβ/δ, and PPARγ, which can be used as indicators of fatty acids and fatty derivatives, playing important roles in lipid and energy processes [8]. In particular, PPARα is highly expressed in metabolically active tissues, such as the liver, heart, and muscle and brown adipose tissues [9]. In the liver, PPARα regulates peroxisomal β-oxidation, mitochondrial β-oxidation, and microsomal ω-oxidation and thus plays a significant role in the occurrence and development of NAFLD [10].

Additionally, studies have shown that the PPARα ligand can effectively lower blood lipid levels, increase insulin sensitivity, and reduce hepatic steatosis [11–13]. In contrast, the loss of PPARα results in starvation-induced accumulation of fat in the liver that cannot be degraded, increasing fatty liver conditions [14, 15]. In addition, PPARα activation can reduce NASH formation from NAFLD by suppressing inflammatory responses [16]. NASH seriousness, visceral adiposity, and insulin resistance were negatively correlated with human liver PPARα gene expression while adiponectin was positively related. A significant correlation has been found between increased expression of PPARα, but not PPARβ/δ or PPARγ, and histological improvement in the obese patients with NAFLD [17]. Based on these data, PPARα is a potential therapeutic target for NASH.

Several reports have studied the expression of human PPARα target genes; however, due to the difficulty of collecting biological samples from healthy subjects, these studies have been performed on small scales that are not representative. Blood is used as a sample source for gene extraction due to its accessibility and minimal harm to volunteers during the collection process. In humans, 20,000–25,000 genes are protein-coding genes, and 66–82% of which are expressed in human blood cells [18]. According to *Cruieiras* et al., assessment of gene expression profiles of peripheral blood mononuclear cells (PBMCs) is an important diagnostic tool for developing therapeutic strategies for obese patients [19]. The present study focused on studying the function of PPARα in the formation of NAFLD through the observation of changes in the expression of PPARα target genes in PBMCs.

## Patients and methods

### Patients

Permission of this study was granted by the Ethics Committee of Human Research at the Medical Examination Center, Shandong University Qilu Hospital, China. All subjects gave their written informed consent to participate in this study, which was approved by the Ethics Committee of Qilu Hosptial of Shandong University (Qingdao), (KYLL-2017031).

The present study consisted of 73 Chinese Han volunteers between 22 and 68 years of age. The selected individuals were divided into three groups according to liver ultrasound and body mass index (BMI), including the control group (CN, *n* = 30; BMI < 30.0 kg/m^2^; normal liver ultrasound), the fatty liver without obesity group (NF, *n* = 21; BMI < 30.0 kg/m^2^; fatty liver tested by ultrasound), and the fatty liver with obesity group (OF, *n* = 22; BMI ≥ 30 kg/m^2^; fatty liver tested by ultrasound). All three groups had absent alcohol consumption (< 20 g/d).

For the initial assessment, individuals answered a standard questionnaire that included personal information about their diets, physical activity, medication used, smoking habits, and family history of chronic diseases. Then, weight, height, abdominal circumference, and blood pressure measurements were recorded. During this evaluation, venous blood samples were collected after 10-h overnight fasting, and BMIs were calculated based on the height and weight of each individual.

*Inclusion criteria*: patients newly diagnosed with non-alcoholic fatty liver disease (NAFLD).

*Exclusion criteria*: subjects with tobacco use habits, chronic use of medications for diabetes mellitus or dyslipidemia, and the diagnosis of chronic medical conditions (e.g., diabetes, hemophilia, heart disease, anemia, and gastrointestinal disease).

### Biochemical blood measurements

Peripheral venous blood (5 mL) was collected following an overnight fasting to measure aspartate aminotransferase (AST), alanine aminotransferase (ALT), r-glutamyl transpeptidase (r-GT), alkaline phosphatase (ALP), total cholesterol (TC), triglyceride (TG), high-density lipoprotein cholesterol (HDLc) and low-density lipoprotein cholesterol (LDLc), free fatty acids (FFA), glucose (GLU), and insulin (INS). These substances were examined using an automatic biochemistry analyzer (Roche Diagnostics GmbH, Germany), and homeostasis model assessment for insulin resistance (HOMA-IR) was calculated from fasting insulin and glucose levels [20].

### Isolation of peripheral blood mononuclear cells (PBMCs)

Immediately after blood collection, the PBMCs were isolated by gradient centrifugation with the Histopaque (density: 1.077, Sigma-Aldrich, Inc.) and washed with a 1× PBS-buffer.

### Total RNA extraction

Total PBMCs RNA was isolated from all samples with Trizol LS® (cat. 15,596–026, Invitrogen), according to the manufacturer’s protocol. The isolated RNA samples were quantified by a spectrophotometer (Titertek-Berthold Colibri, Germany).

### cDNA synthesis

cDNA was synthesized using the UEIris reverse transcription real-time polymerase chain reaction (RT-PCR) system. Total RNA (500 ng/ml in a final volume of 2 μl) was added to a mixture (10 μl) containing RNase-free water, 10 × DNase I buffer, and DNase I. This mixture was placed into a thermal cycler (Bioer Life Express, China) at 37 °C for 10 min, before 1 μl of 50 mM EDTA was added to the mix. This mixture was heated to 65 °C for 10 min to deactivate DNase I; then, UEIris RT MasterMix (5×) RNase-free water was added to the mix (20 μl). This mix was placed into the thermal cycler, and a reverse transcription reaction was facilitated to produce cDNA at 55 °C for 30 min, with a final step conducted for 10 s at 85 °C.

### Conventional PCR

After cDNA was synthesized, a conventional PCR was conducted to evaluate its quality, and β-actin was found to be stably expressed under the described laboratory conditions in PBMCs. After quantification using a spectrophotometer (Titertek-Berthold Colibri, Germany) and confirmation of cDNA quality by electrophoresis (40 V, 30 mA for 60 min) on a 2% agarose gel (UltraPure, cat. 15,510,019, Invitrogen), a second PCR reaction was performed to assess the efficiencies of the forward and reverse primers of the following genes: pyruvate dehydrogenase kinase isoform 4 (PDK4), carnitine palmitoyltransferase 1A (CPT1A), carnitine palmitoyltransferase 1B (CPT1B), acetyl-coenzyme A dehydrogenase 2 (ACAA2), solute carrier family 25 (carnitine/acylcarnitine translocase) member 20 (SLC25A20), and acyl-coenzyme A dehydrogenase long chain (ACADVL). Primers used in this study are shown in Table 1.

**Table 1:** Primer sequences for the RT-PCR

Gene	Primers	Sequence (5’-3’)	Size (bp)
β-actin	Sense	GGCTGTATTCCCCTCCATCG	154
	Antisense	CCAGTTGGTAACAATGCCATGT	
Solute carrier family 25 (carnitine/acylcarnitine translocase) member 20 (SLC25A20)	Sense	GGGGTCACTCCCATGTTTG	135
	Antisense	TGTGGTGAATACGCCAGATAAC	
Pyruvate dehydrogenase kinase isoform 4 (PDK4)	Sense	GGAGCATTTCTCGCGCTACA	117
	Antisense	ACAGGCAATTCTTGTCGCAAA	
Peroxisome proliferators activated receptors α (PPARα)	Sense	ATGGTGGACACGGAAAGCC	124
	Antisense	CGATGGATTGCGAAATCTCTTGG	
Carnitine palmitoyltransferase 1B (CPT1B)	Sense	GCGCCCCTTGTTGGATGAT	112
	Antisense	CCACCATGACTTGAGCACCAG	
acetyl-coenzyme A dehydrogenase 2 (ACAA2)	Sense	CTGCTCCGAGGTGTGTTTGTA	105
	Antisense	GGCAGCAAATTCAGACAAGTCA	
Acyl-coenzyme A dehydrogenase, long chain (ACADVL)	Sense	ACAGATCAGGTGTTCCCATACC	114
	Antisense	CTTGGCGGGATCGTTCACTT	
Carnitine palmitoyltransferase 1A (CPT1A)	Sense	TCCAGTTGGCTTATCGTGGTG	98
	Antisense	TCCAGAGTCCGATTGATTTTTGC	

### Quantitative real-time polymerase chain reaction (qPCR)

A Q-PCR was utilized to assess the mRNA expression levels of target genes. Reactions were performed in the SLAN-96S using the Chromo 4 Detection System (MJ Research Inc.) and the Platinum SYBR Green qPCR Supermix-UDG (cat. S-2008, US Everbright Inc.). PCR amplification conditions were as follows: one cycle at 95 °C for 2 min, 40 cycles at 95 °C for 5 s, one cycle at 60 °C for 15 s, and elongation at 72 °C for 25 s. Each cDNA sample was analyzed in triplicate for each gene. The relative expression ratio of mRNA was calculated using the equation 2^−ΔΔCt^, where −ΔCt refers to the difference between the numbers of cycles (Ct) of the target genes and the endogenous control.

### Statistical analysis

The analysis was performed using the statistical software SPSS 17.0. Values were expressed as the mean ± standard deviation (SD) whenever applicable. The differences between the groups were verified using the one-way ANOVA. The correlation of PPARα target genes with main clinical parameters in the OF group was determined using the Spearman rank test. A *P*-value of less than 0.05 was considered statistically significant.

## Results

### Clinical and biochemical characteristics

The characteristics of the studied individuals are presented in the Table 2. The patients in all three sample groups were close in age (*P* > 0.05). In the OF group, BMI, waist, and systolic blood pressure (SBP) were all significantly higher than those in the CN group and NF group (*P* < 0.01). Diastolic blood pressure (DBP) in both NF and OF groups was higher than that in the CN group (*P* < 0.05). Based on these data, fatty liver patients have increased blood pressure, while obese patients have higher chances of having increased SBP.

**Table 2:** Clinical and biochemical characteristics of the CN, NF and OF groups

	CN group	NF group	OF group	F	P
Age, years	38.63 ± 12.68	46.31 ± 11.61	39.05 ± 11.14	2.59	0.08
BMI,^†^ Kg/m^2^	24.31 ± 4.14	25.45 ± 2.82	31.78 ± 2.17**ΔΔ	30.95	0.00
Waist,^†^ cm	85.45 ± 14.67	91.26 ± 10.78	112.89 ± 6.76**ΔΔ	31.81	0.00
SBP,^†^ mm Hg	120.59 ± 15.74	138.79 ± 18.58**	141.37 ± 18.90**ΔΔ	8.55	0.001
DBP,^†^ mm Hg	73.36 ± 12.21	80.74 ± 8.52*	87.26 ± 13.48**	7.32	0.001
ALT,^†^ U/L	24.09 ± 11.05	29.10 ± 13.34	49.74 ± 36.95**ΔΔ	6.87	0.002
AST,^†^ U/L	18.77 ± 4.30	20.63 ± 6.90	27.95 ± 14.61**Δ	5.23	0.008
ALP,^†^ U/L	66.63 ± 14.53	79.26 ± 28.35*	74.68 ± 13.65*	2.15	0.126
rGT,^†^ U/L	22.64 ± 10.52	38.37 ± 28.40	83.58 ± 61.12*	2.35	0.105
TC,^†^ mmol/L	4.66 ± 0.88	5.52 ± 1.00**	5.02 ± 0.91	4.38	0.017
TG,^†^ mmol/L	1.42 ± 0.68	2.21 ± 1.55*	1.73 ± 0.66	3.05	0.055
HDLc,^†^ mmol/L	1.18 ± 0.20	1.15 ± 0.17	2.05 ± 2.64	2.31	0.109
LDLc,^†^ mmol/L	1.93 ± 0.46	2.53 ± 0.56**	2.18 ± 0.48Δ	7.51	0.001
FFA,^†^ mEq/L	0.94 ± 0.24	1.15 ± 0.60	1.06 ± 0.31	1.42	0.25
Glu,^†^ mmol/L	4.86 ± 0.55	5.72 ± 1.07**	5.59 ± 1.01*	4.97	0.01
INS,^†^ uIU/ml	8.11 ± 6.17	13.25 ± 8.02*	14.86 ± 8.01**	4.76	0.012
HOMA-IR,^†^	1.78 ± 1.41	3.52 ± 2.48**	3.69 ± 2.16**	5.52	0.006

^†^Data were expressed as the mean ± SD of 30 people in the CN group, 21 people in the NF group, and 22 people in the OF group. Statistically significant differences between groups were determined using ANOVA (*p < 0.05, **p < 0.01, ***p < 0.001 vs the CN group; ^Δ^*p* < 0.05, ^ΔΔ^*p* < 0.01 vs the NF group)

*ALP* Alkaline phosphatase, *ALT* Alanine transaminase, *AST* Aspartate transaminase, *BMI* body mass index, *DBP* diastolic blood press, *FFA* free fatty acids, *Glu* Glucose, *HDLc* High-density lipoprotein cholesterol, *HOMA-IR* Homeostasis model assessment of insulin resistance, *INS* Insulin, *LDLc* Low-density lipoprotein cholesterol, *SBP* Systolic blood press, *SD* standard deviation, *TC* Total cholesterol, *TG* Triacylglycerol, *rGT* gamma-glutamyl transpeptidase

The four hepatic metabolic enzymes, ALT, AST, ALP, and r-GT were higher in the OF group compared with the CN group (*P* < 0.05), and the ALT and AST were significantly higher in the OF group when comparing to the NF group (*P* < 0.05). Increased serum AST and ALT levels are used as markers for liver damage, and here, it was proved that OF patients had increased liver function damage. Besides, NF patients had significantly higher levels of TC, TG, and LDLc than the CN patients. In contrast, there was an upward trend in blood lipids of the OF group relative to the CN group without statistical difference, indicating that the disturbances in lipid metabolism were more serious in NF patients, while the fatty liver formation in the OF group might result from other factors, such as adipocytokines. Further, the fasting blood glucose levels, insulin levels, and HOMA-IR were significantly higher in the NF and OF groups than those in the CN group (*P* < 0.05); there was no significant difference between the NF and OF groups; therefore, fatty liver patients have significant IR that does not depend on obesity.

### Gene expression

**Fig. 1:**
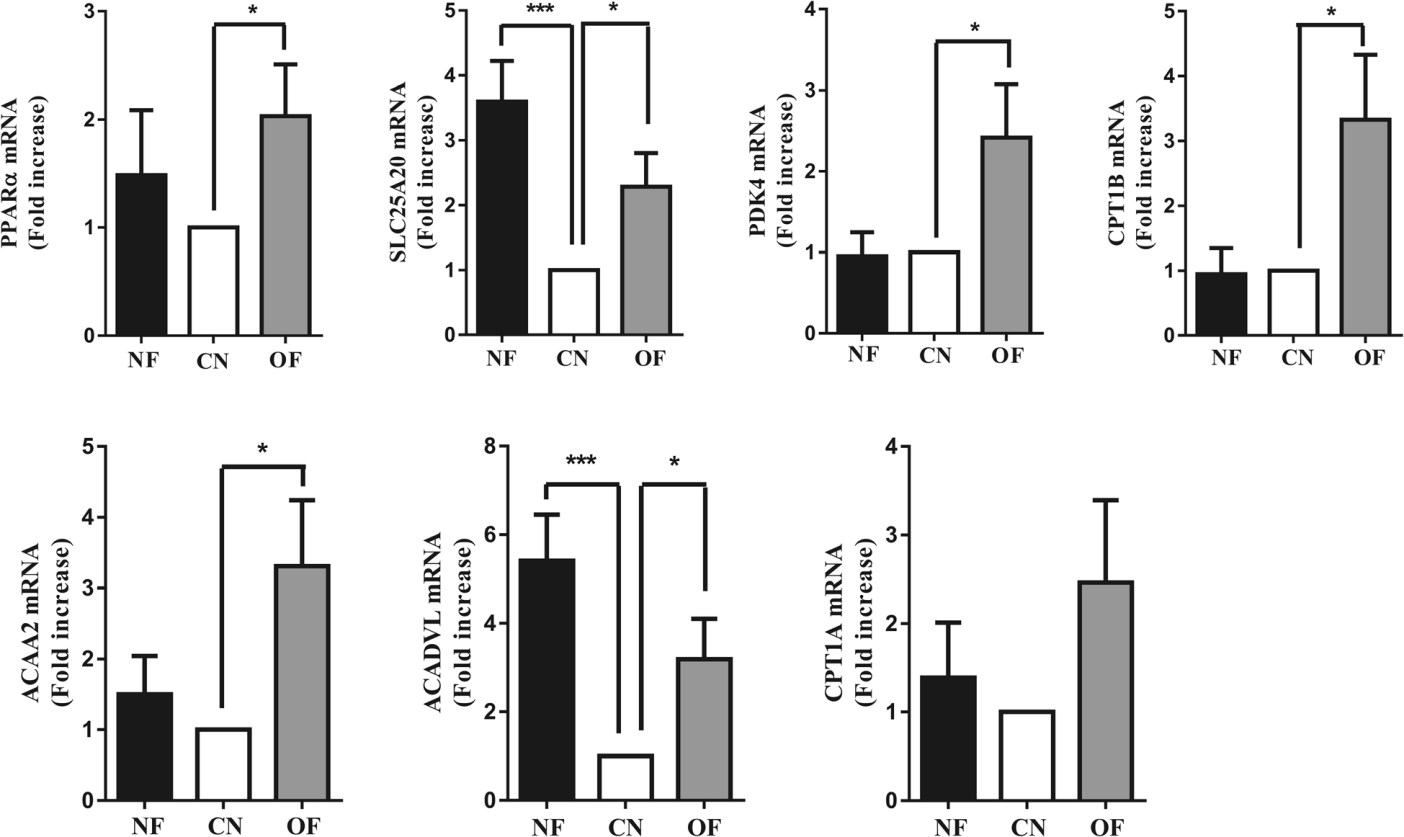
Gene expression of PPARα target genes in PBMCs. Data were expressed as the mean ± SD of 30 people in the CN group, 21 people in the NF group, and 22 people in the OF group. Statistically significant differences between groups were determined using ANOVA; *p < 0.05, **p < 0.01, ***p < 0.001 vs the CN group

The PPARα expression was significantly higher in the OF group than that in the CN group (*P* < 0.05); the expression of PPARα target genes (SLC25A20, PDK4, CPT1B, ACAA2, and ACADVL) in the OF group was 2.03–3.31 times more than that in the CN group (*P* < 0.05), while CPT1A expression was similar in the CN group and OF group (*P* > 0.05) (Fig. 1). In the NF group, the SLC25A20 and ACADVL expression were significantly higher than that in the CN group (*P* < 0.05) (Fig. 1).

The correlation of PPARα and its target genes with primary clinical parameters in the OF group is presented in the Table 3. There was a negative correlation between PPARα target gene expression and abdominal circumference (AC), BMI, DBP. SLC25A20 was negatively correlated with insulin and HOMA-IR; ACADVL was negatively correlated with blood glucose and HOMA-IR; PPARα was positively correlated to blood glucose, insulin, and HOMA-IR; ACAA2 was positively correlated with insulin and HOMA-IR; CPT1A was positively correlated with HOMA-IR.

**Table 3:** Correlation of PPARα and its target genes with main clinical parameters in OF group

OF group		SLC	PDK4	PPARα	CPT1B	ACAA2	ACADVL	CPT1A
AC,^†^	Pearson Correlation	.112	−.436*	−.383	−.411*	−.131	.303	−.403*
	Significant (unilateral)	.324	.031	.053	.040	.297	.104	.044
BMI,^†^	Pearson Correlation	.360	−.532**	−.577**	−.512*	−.499*	.450*	−.549**
	Significant (unilateral)	.065	.010	.005	.012	.015	.027	.007
SBP,^†^	Pearson Correlation	−.065	−.204	−.175	−.233	−.035	−.051	−.285
	Significant (unilateral)	.396	.201	.236	.168	.443	.417	.118
DBP,^†^	Pearson Correlation	.245	−.573**	−.561**	−.628**	−.395*	.270	−.677**
	Significant (unilateral)	.156	.005	.006	.002	.047	.132	.001
ALT,^†^	Pearson Correlation	−.373	−.486*	−.365	−.387	−.228	−.177	−.382
	Significant (unilateral)	.058	.018	.062	.051	.173	.235	.053
GLU,^†^	Pearson Correlation	−.264	.425*	.484*	.388	.382	−.530**	.385
	Significant (unilateral)	.137	.035	.018	.050	.053	.010	.052
INS,^†^	Pearson Correlation	−.432*	.224	.422*	.291	.441*	−.349	.326
	Significant (unilateral)	.032	.178	.036	.114	.029	.071	.086
HOMA -IR,^†^	Pearson Correlation	−.444*	.342	.546**	.377	.538**	−.445*	.424*
	Significant (unilateral)	.028	.076	.008	.056	.009	.028	.035

^†^In the OF group, the correlation between PPARα target genes and primary clinical parameters were determined using the Spearman rank test (*p < 0.05,**p < 0.01)

*AC* Abdominal circumference, *ALT* Alanine transaminase, *BMI* body mass index, *DBP* diastolic blood press, *Glu* Glucose, *HOMA-IR* Homeostasis model assessment of insulin resistance, *INS* Insulin, *SBP* Systolic blood press

## Discussion

PPARα is a key factor involved in the cellular metabolic response, and its gene expression was examined through liver biopsy among NAFLD patients, showing significantly lower levels of expression for increasing severity of NASH [17]. In this study, high blood pressure and IR were found in all NAFLD patients, with fatty liver, obesity, and increased liver damage. The PPARα gene expression was found to be increased in the PBMCs of the OF group, having a negative correlation to BMI, DBP and a positive correlation to blood glucose, insulin, HOMA-IR. Thus, these findings suggest that PPARα gene expression follows the same trend as IR in human liver tissue.

Increasing evidence suggests that PPARα plays a main role in the management of obesity, especially central obesity associated with IR syndromes, given its involvement in the regulation of lipid metabolism and inflammation. Stimulation of β-oxidation by PPARα activation is a suggested mechanism for decreasing tissue lipid content to prevent lipid accumulation [21]. In an animal model, PPARα activation was shown to have a beneficial effect on insulin sensitivity by reducing plasma TG levels and adiposity [16]. In humans, fibrate activation of PPARα has been shown to increase the circulating levels of atheroprotective HDLc, to lower plasma levels of TG and FFA, and to improve overall atherogenic plasma lipid profiles while exerting beneficial effects on inflammation, IR, and metabolic syndrome [22–26]. In our study, NF patients had significantly higher levels of TC, TG, and LDLc than the CN patients. It has been reported that nutraceuticals and functional food ingredients that are beneficial to vascular health reduce the dyslipidemia-induced cardiovascular risk possibly by decreasing 7α-hydroxylase, increasing fecal excretion of cholesterol or decreasing the secretion of very low-density lipoprotein [27]. These findings suggest that nutraceuticals and functional food ingredients that are beneficial to liver health might reduce the incidence of NAFLD.

Through testing PPARα target gene expression in PBMC samples and conducting a correlation analysis, we found that the OF group had a 2.46-time increase in CPT1A gene expression compared to the CN group, which was positively related to HOMA-IR. Blood glucose levels decreased concomitantly with a decrease in cellular glycolysis, and CPT1A was activated, permitting the mitochondrial entry of FFA [28, 29]. However, as CPT1A is a crucial enzyme involved in FFA catabolism within mitochondria in metabolically active tissue, the up-regulation of CPT1A gene expression in PBMCs may indicate an increased β-oxidation rate to meet energy demands [21]. During fasting, increased CPT1A gene expression is an essential metabolic response to maintain cellular lipid balance in liver tissues, which increases FFA absorption and utilization [30]. In the present study, CPT1B was found to be increased and have a negative correlation with AC and BMI, however, the exact mechanism remains unclear.

PDK4 is a gatekeeper regulator for glucose metabolism [31]. According to the results in animal experiments, the mRNA and protein levels of PDK4 are elevated in livers of diabetic, fasted, and IR animals [32, 33]. Furthermore, PDK4 knock-out mice have been found to have lower blood glucose levels, better glucose tolerance, and greater insulin sensitivity compared to wild-type mice [34]. In differentiated cultured human myotubes, agonist (GW7647)-mediated activation of PPAR-α results in increased fatty acid oxidation [35, 36], diminished accumulation of TG [35], and upregulation of PDK4 [37]. In a recent study, the mRNA and protein levels of PDK4 were upregulated in NASH livers, indicating that PDK4 potentially contributes to the hepatic steatosis of NASH via regulation of several signaling pathways [38]. PDK4 phosphorylates and inactivates the pyruvate dehydrogenase complex during fasting, thereby decreasing glucose oxidation [39, 40]. We for the first time confirm that PDK4 up-expression in the PBMCs of the OF group negatively correlates to AC, BMI and positively correlates to blood glucose. Thus, PDK4 may be a new therapeutic agent for NAFLD.

Several studies have reported SLC25A20, ACADVL, and ACAA2 genes in fatty liver PBMCs; in our study, an increase in the mean fold change of the ACADVL gene among obese individuals was observed. This gene codes for an enzyme that controls a critical point in the supply of electrons to the respiratory chain and is responsible for catalyzing the first β-oxidation step [41]. CPT1A and ACADVL are lipid oxidation markers and were observed to be decreased in newborn puppies compared to obese dams [42], while another report showed that PPARα and ACAA2 are closely associated with NASH [43]. In this study, it was found that blood glucose, insulin level, and HOMA-IR were negatively related to SLC25A20, ACADVL but positively related to ACAA2 in the OF group, although further experiments are required to confirm these results.

There have been several limitations of this study. The sample size is relatively small. A further study with larger number of samples is required in the future work. Besides, a multivariate regression analysis should be performed in order to evaluate the role of confounding factors on final results.

## Conclusion

In summary, the effects of PPARα gene were studied by analyzing its target gene expression in the PBMCs of NAFLD patients. PPARα target genes were found to be negatively correlated with AC, BMI, and DBP in OF patients, while SLC25A20 and ACADVL were negatively correlated with blood glucose, insulin level, and HOMA-IR, and PPARα, ACAA2, and CPT1A were positively related to the same variables. These results reveal the up-expression of PPARα target genes in NAFLD patients’ PBMCs, possibly affecting β-oxidation and IR.

## Abbreviations

AC: Abdominal circumference
ACAA2: Acetyl-coenzyme A dehydrogenase 2
ACADVL: Acyl-coenzyme A dehydrogenase 2 long chain
ALP: Alkaline phosphatase
ALT: Alanine aminotransferase
AST: Aspartate aminotransferase
BMI: Body mass index
CN: Control group
CPT1A: Carnitine palmitoyltransferase 1A
CPT1B: Carnitine palmitoyltransferase 1B
Ct: Cycles
DBP: Diastolic blood pressure
FFA: Free fatty acids
GLU: Glucose
HDLc: High-density lipoprotein cholesterol
HOMA-IR: Homeostasis model assessment for insulin resistance
INS: Insulin
LDLc: Low-density lipoprotein cholesterol
NAFLD: Non-alcoholic fatty liver disease
NASH: nonalcoholic steatohepatitis
NF: Non-obese group
OF: Obese group
PBMCs: Peripheral blood mononuclear cells
PDK4: Pyruvate dehydrogenase kinase isoform 4
PPARs: Peroxisome proliferator-activated receptors
PPARα: Peroxisome proliferator-activated receptor alpha
qPCR: Quantitative real-time polymerase chain reaction
r-GT: r-glutamyl transpeptidase
RT-PCR: Reverse transcription real-time polymerase chain reaction
SBP: Systolic blood pressure
SD: Standard deviation
SLC25A20: Solute carrier family 25 (carnitine/acylcarnitine translocase) member 20
TC: Total cholesterol
TG: Triglyceride
